# The prognostic value of sialylation-related long non-coding RNAs in lung adenocarcinoma

**DOI:** 10.1038/s41598-024-59130-3

**Published:** 2024-04-17

**Authors:** Beiru Wang, Chengyu Hou, Xiang Yu, Jiaxin Liu, Jiyong Wang

**Affiliations:** 1 First Clinical Medical College, Guangzhou University of Chinese Medicine, Guangzhou, 510405 Guangdong China; 2 Department of Thoracic Surgery, The First Affiliated Hospital of Guangzhou University of Chinese Medicine, Guangzhou, 510405 Guangdong China

**Keywords:** Sialylation, Long non-coding RNA, Lung adenocarcinoma, Prognostic model, Lung cancer, Tumour biomarkers

## Abstract

There has been increasing interest in the role of epigenetic modification in cancers recently. Among the various modifications, sialylation has emerged as a dominant subtype implicated in tumor progression, metastasis, immune evasion, and chemoresistance. The prognostic significance of sialylation-related molecules has been demonstrated in colorectal cancer. However, the potential roles and regulatory mechanisms of sialylation in lung adenocarcinoma (LUAD) have not been thoroughly investigated. Through Pearson correlation, univariate Cox hazards proportional regression, and random survival forest model analyses, we identified several prognostic long non-coding RNAs (lncRNAs) associated with aberrant sialylation and tumor progression, including LINC00857, LINC00968, LINC00663, and ITGA9-AS1. Based on the signatures of four lncRNAs, we classified patients into two clusters with different landscapes using a non-negative matrix factorization approach. Collectively, patients in Cluster 1 (C1) exhibited worse prognoses than those in Cluster 2 (C2), as well as heavier tumor mutation burden. Functional enrichment analysis showed the enrichment of several pro-tumor pathways in C1, differing from the upregulated Longevity and programmed cell death pathways in C2. Moreover, we profiled immune infiltration levels of important immune cell lineages in two subgroups using MCPcounter scores and single sample gene set enrichment analysis scores, revealing a relatively immunosuppressive microenvironment in C1. Risk analysis indicated that LINC00857 may serve as a pro-tumor regulator, while the other three lncRNAs may be protective contributors. Consistently, we observed upregulated LINC00857 in C1, whereas increased expressive levels of LINC00968, LINC00663, and ITGA9-AS1 were observed in C2. Finally, drug sensitivity analysis suggested that patients in the two groups may benefit from different therapeutic strategies, contributing to precise treatment in LUAD. By integrating multi-omics data, we identified four core sialylation-related lncRNAs and successfully established a prognostic model to distinguish patients with different characterizations. These findings may provide some insights into the underlying mechanism of sialylation, and offer a new stratification way as well as clinical guidance in LUAD.

## Introduction

Lung adenocarcinoma (LUAD), a predominant subtype of non-small cell lung cancer (NSCLC), is a prevalent malignant cancer worldwide, with the highest mortality and the most new cases compared to other solid tumors in China^1^. Extensive efforts have been devoted to gaining insights into the underlying mechanisms during tumor progression in LUAD, with particular emphasis on profiling genomic alterations. Despite great advances that have been made in targeted therapy, the 5-year survival rate is relatively low, and drug resistance inevitably occurs during treatment. Subsequently, studies focused on epigenetic modification were gradually carried out, such as phosphorylation, methylation, and glycosylation^2^. Post-translational modifications (PTMs) occur in more than 50% of proteins and directly influence the function of proteins in many fundamental biological processes. Of these PTMs, glycosylation characterized by complex structures and sites amounts to almost half and can be observed in both membrane-bound and secreted proteins^3^. Of note, the thick layer of glycans covering cell surfaces is responsible for the communication with extracellular environment and exhibits distinct characteristics between healthy and cancer cells^4^, highlighting the enormous potential in diagnosis and drug discovery. Recent works have suggested that the levels of glycosylation may serve as prognostic predictors in NSCLC^5^.

Sialylation, a subtype of glycosylation, refers to the process of containing the covalent addition of sialic acid to the terminal glycans of glycoproteins and glycolipids. The level of sialylation is mainly regulated by activities of sialyltransferase enzymes. Four groups of sialyltransferase, including ST6GAL, ST3GAL, ST6GALNAC and ST8SIA, can yield distinct sialyation patterns in cells^6,7^. Due to the involvement in tumor progression, metastasis, immune evasion, and therapeutic resistance, hypersialylation and increased activities of sialyltransferases have been recognized as novel cancer hallmarks recently^8,9^. Specifically, previous studies performed on cancer cells revealed that hypersialylated integrins decreased cell adhesion and increased cell migration, further inducing metastatic phenotype^10,11^. For instance, through upregulating sialyltransferases content, oncogenes Ras and c-Myc respectively increased the sialylation levels of β1 integrin and E-selectin ligand, further inducing epithelial-mesenchymal transition (EMT) process in cancer cells^12,13^. Additionally, one of the reasons why cancer cells can evade immune surveillance is that the elevated levels of sialic acids located in the outermost layer of cancer cells, sialylation of MHC class I–related chain A, sialylated tumor-associated carbohydrate antigens limit the anti-tumor immune response^14^. These findings provide some valuable insights into the potential role of sialylation-related molecules as biomarkers in clinical management. Notably, the developed antibodies of sialic acid-binding immunoglobulin-like lectins and inhibitors of sialyltransferases may bring new hope for therapeutic intervention^4^.

Recent studies performed on LUAD have reported the establishment of novel cancer hallmarks, such as the upregulation of sialyltransferases (STs) and hypersialylation of tumor cell surfaces. Through the comprehensive investigation of ST3GAL family, researchers found that silent ST3GAL6 and ST3GAL6-AS1 promoted malignant cells invasiveness through activating EGFR/MAPK signaling pathways and were associated with poor prognoses in LUAD patients^15^. As the field of sialylation in lung cancer continues to develop, an increasing number of sialylation-related biomarkers have been identified, demonstrating associations with tumor progression and metastasis, including ST3Gal-IV^16^, ST8SIA2^17^, P-3FAX-Neu5Ac^18^, ST6GalNAc1^19^, Siglec-9^20^, Siglec-15^21^, ST6Gal-I^22^. Remarkably, preclinical studies have shown the survival benefits of ST inhibitors in lung cancer. For instance, AL10, a pan-ST inhibitor targeting integrins that overexpresses α2,3-ST, effectively impedes the migration of lung cancer cell lines in vitro and in vivo experiments, exhibiting good safety profiles^23^. Unfortunately, a considerable portion of ST inhibitors suffer from poor membrane permeability, resulting in impaired drug efficacy. The lack of clinical trials further limits the widespread application, requiring the identification of novel therapeutic targets.

Long non-coding RNAs (lncRNAs), with lengths longer than 200 nucleotides, are important regulators mediating various biological processes, including transcriptional regulation, cell proliferation and differentiation, epigenetic modification, and metabolic reprogramming. Previous studies have revealed that lncRNAs are involved in dysregulated sialylation processes and pathogenesis in cancers. For instance, downregulated lncRNA MEG3 influences the sialylation of EGFR by positively regulating ST3Gal1, thereby inhibiting the activation of PI3K-AKT pathway in renal cell carcinoma^24^. Another study suggested that upregulated lncRNA ST3GAL6-AS1 promoted the invasion of multiple myeloma by increasing the expression of sialyltransferase ST3GAL6. Additionally, sialylation-related lncRNAs have shown prognostic value. In colorectal cancer (CRC), researchers successfully constructed a prognostic model based on the signatures of seven sialylation-related lncRNAs, which showed reliable performance in classifying patients into different clinical outcomes, immune infiltrating levels, and drug responses^25^. These findings highlight the significance of sialylation-related lncRNAs in cancer biology and suggest their potential as prognostic markers and therapeutic targets. Given the large number of lncRNAs whose biological functions remain unknown, it is plausible that numerous potential lncRNAs are involved in sialylation and siglec interactions. In LUAD, a systemic analysis is necessary to characterize the associations of lncRNAs with tumor progression or patient survival, aiming to provide some insights for the discoveries of novel therapeutic targets.

In this study, four prognostic lncRNAs related to sialylation were identified based on the data from The Cancer Genome Atlas (TCGA) and GSE31210, and the regulatory network was profiled. Subsequently, we successfully constructed a prognostic model and obtained two clusters characterized by different clinical outcomes. The underlying mechanisms were described by exploring the differences in tumor mutation burden (TMB), and immune and metabolic microenvironment. The findings of drug responses indicated that patients with discrepant signatures may benefit from different treatments. Taken together, these results showed that the signature of four sialylation-related lncRNAs can serve as a prognostic predictor, providing some insights into the clinical management for patients with LUAD.

## Results

### Identification of prognostic sialylation-related lncRNAs

**Figure 1:**
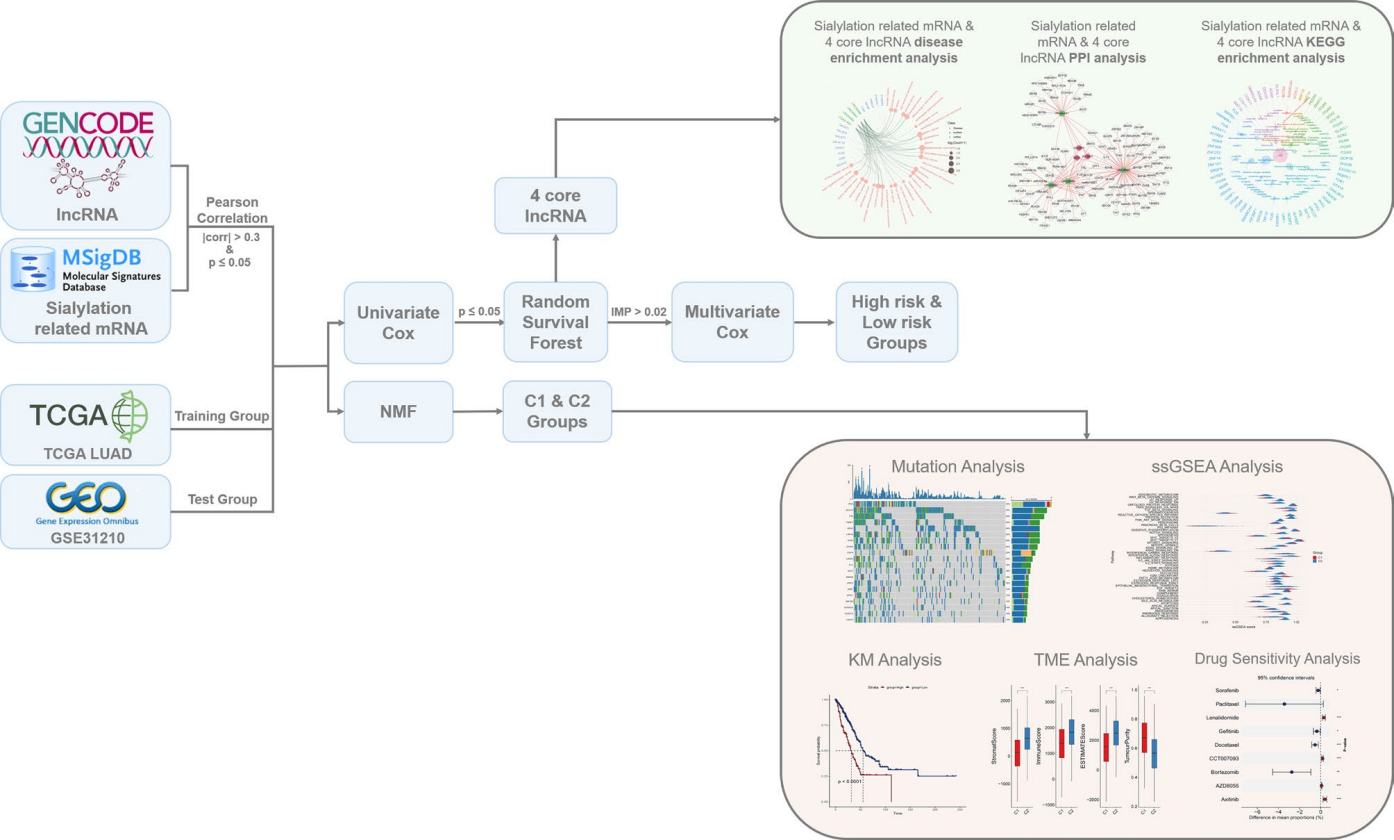
Flow chart of this study.

**Figure 2:**
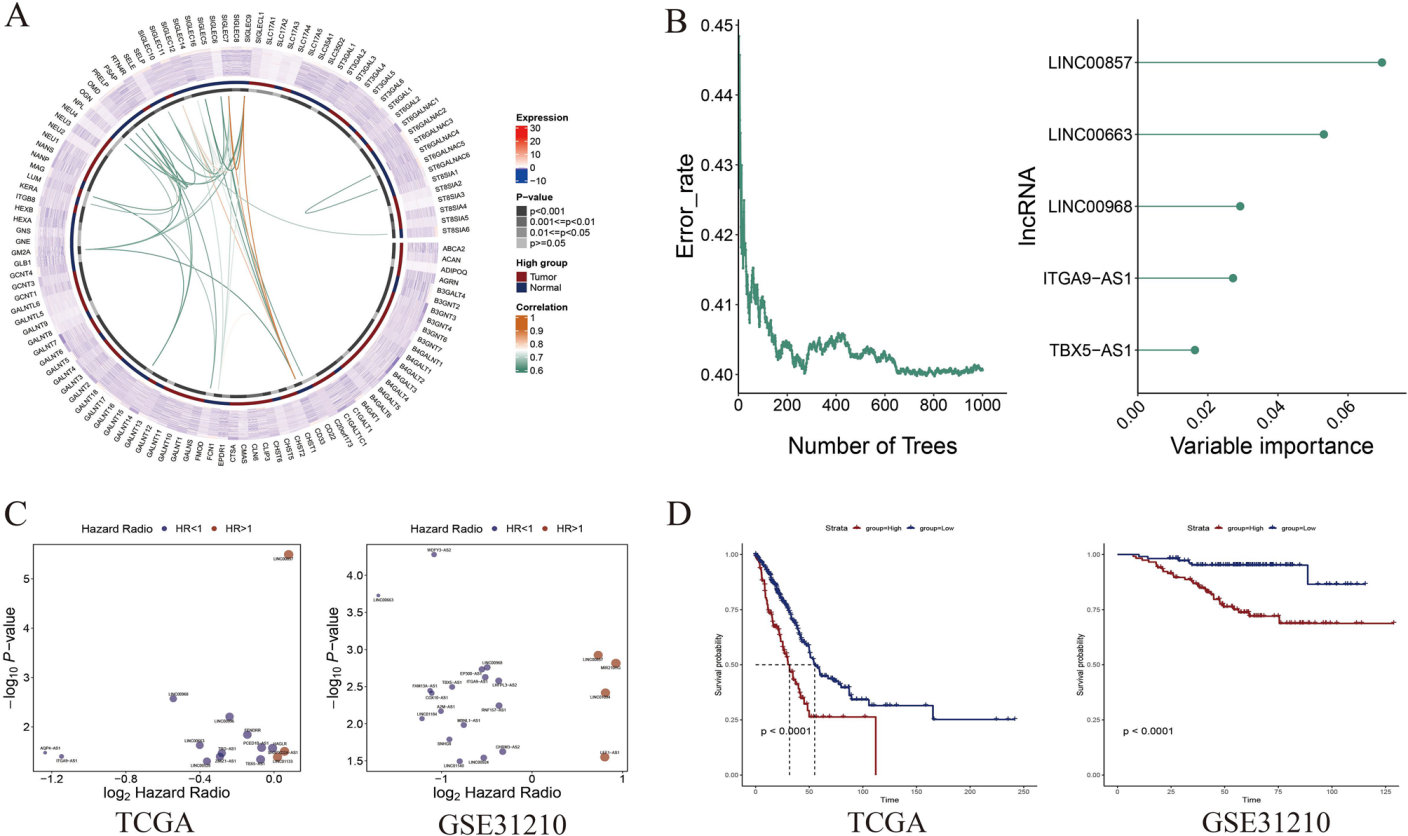
The identification of prognostic sialylation-related lncRNAs in LUAD. (**A**) The expression profiles of sialylation-related genes between tumors and adjacent normal tissues. Heatmap presents the expression levels of sialylation-related genes across all samples. Second circle from the inside out, with alternating red and blue segments, indicates the gene is highly expressed in tumor group or normal group. Red and blue segments represent the high expression levels in Tumor and Normal groups, respectively. First gray-black circle from the inside out visualizes the significance result of expression level for each gene between tumor and normal samples. Inner lines represent the correlation among sialylation-related genes. (**B**) Sialylation-related lncRNAs with the highest scores of variable importance. (**C**) Hazard ratios of important lncRNAs in TCGA-LUAD and GSE31210 datasets. (**D**) Kaplan–Meier survival curves of patients with different risk scores in TCGA-LUAD and GSE31210 datasets.

**Figure 3:**
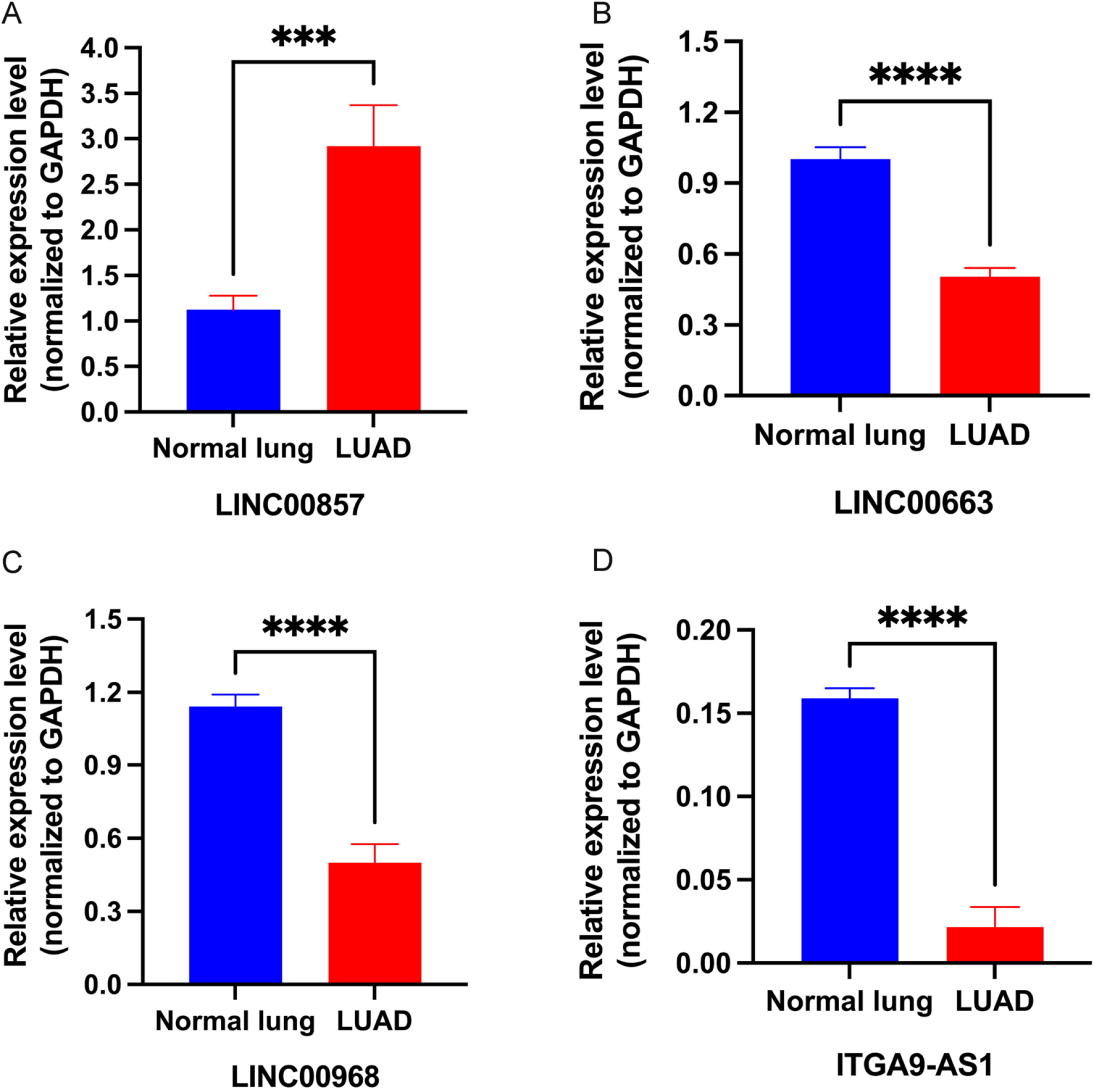
RT-qPCR results of four lncRNAs in tumor and normal tissues of LUAD patients, including (**A**) LINC00857, (**B**) LINC00663, (**C**) LINC00968, and (**D**) ITGA9-AS1.

The process for exploring the prognostic value of sialylation-related lncRNA expression in colorectal cancer is shown in Fig. 1. With 504 tumors and 58 normal tissues collected from TCGA-LUAD, we observed that most sialylation-related genes were differently expressed in two groups of samples, with statistical significance. (Fig. 2A). This finding preliminarily supported the presence of aberrant sialylation processes in intratumor microenvironment. To recognize lncRNAs associated with sialylation, the correlation of lncRNA with sialylation-related mRNA was evaluated. After filtering, 70 sialylation-related lncRNAs shared by TCGA-LUAD and GSE31210 cohorts were utilized for univariate COX regression analysis to assess the potential prognostic roles of these lncRNAs. The overlapped 5 lncRNAs (LINC00857, LINC00663, LINC00968, ITGA9-AS1, and TBX5-AS1) with statistical significance were further employed to perform random forest analysis. Consequently, there were four lncRNAs with the highest variable importance exceeding 0.02, including LINC00857, LINC00663, LINC00968, and ITGA9-AS1 (Fig. 2B). The hazard ratios generated from univariate COX regression analysis suggested that LINC00857 was a risk factor (HR > 1, *P* < 0.05), while LINC00968, LINC00663, and ITGA9-AS1 seemed to play protective roles (HR < 1, *P* < 0.05) in LUAD (Fig. 2C), which were consistent between TCGA-LUAD and GSE31210 data. To validate the findings,we performed reverse transcription quantitative polymerase chain reaction (RT-qPCR) utilizing paired tumor and adjacent tissues from 4 LUAD patients. Results confirmed the overexpression of LINC00857 and the reduced expression of ITGA9-AS1, LINC00663, and LINC00968 (Fig. 3). Based on the risk scores calculated by multivariate COX proportional regression analysis, we divided samples into high- or low-risk groups. Subsequently, Kaplan–Meier survival analysis revealed significantly poor prognoses in high-risk group of TCGA-LUAD, which was consistent with the worse clinical outcomes observed in samples from GSE31210 dataset (Fig. 2D). Taken together, these explorations facilitated us  to focus on the four core lncRNAs and preliminarily demonstrated their involvement in tumorigenesis of LUAD.

### Prediction of interaction network between sialylation-related lncRNA and other molecules

**Figure 4:**
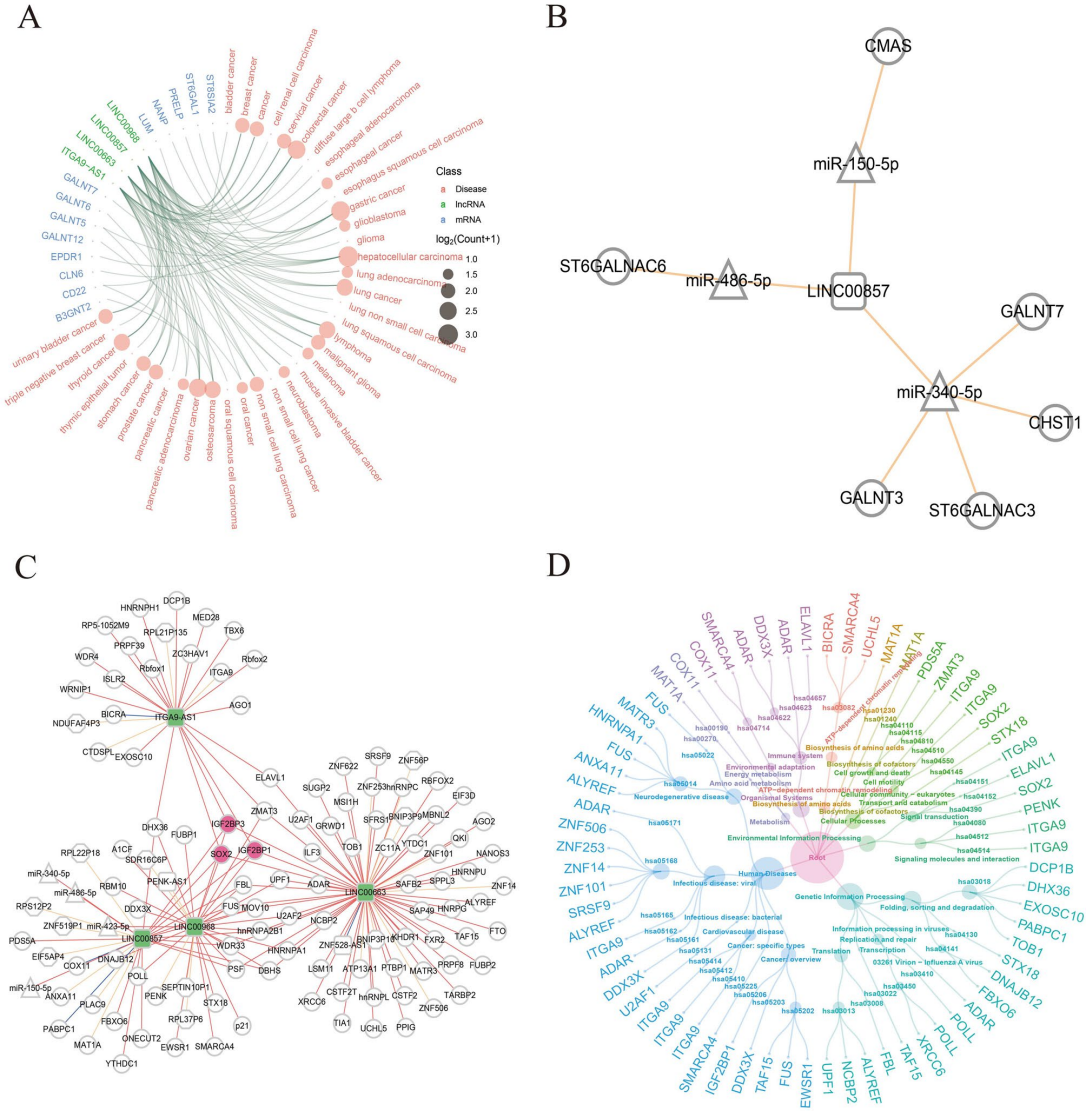
Regulatory networks of sialylation-related lncRNAs. (**A**) Associations between sialylation-related RNAs and cancers. (**B**) Regulatory network mediated by LINC00857 on sialylation-related genes. (**C**) Predicted target genes of the four core lncRNAs. (**D**) Dendrogram of biological pathways involving target genes potentially targeted by lncRNAs.

Based on the known molecular-disease associations in databases, we performed disease enrichment analysis, and the results provided additional evidence for the involvement of these core sialylation-related lncRNAs in cancers (Fig. 4A). To investigate the underlying molecular mechanism, the lncRNA-miRNA interaction pairs were collected from databases, and potential target genes were predicted. As a result, several sialylation-related genes were recognized, and the sialylation processes may be regulated by LINC00857 through interacting with miR-486-5p/ST6GALANC6, miR-150-5p/CMAS, miR-340-5p/GALNT7, miR-340-5p/CHST1, miR-340-5p/ST6GALNAC3, and miR-340-5p/GALNT3 (Fig. 4B). For other three lncRNAs without published relationships with miRNAs, we provided predicted gene networks (Fig. 4C). Functional correlation analysis of target genes suggested that the four lncRNAs may participate in multiple biological processes through influencing genes expressions, such as Protein processing in endoplasmic reticulun, Cysteine and methionine metabolism, Biosynthesis of amino acids, ATP-dependent chromatin remodeling, and Transcriptional misregulation in cancer (Fig. 4D). Endoplasmic reticulun is one of the places where PTM occurred, and FBXO6 and DNAJB12, two predicted targets of LINC00857, were enriched in Protein processing in endoplasmic reticulum (hsa04141) pathway. Of note, several genes, IGF2BP3, IGF2BP1, and SOX2, were uncovered to interact with all the four lncRNAs. Previous studies have reported the overexpression of SOX2 promotes the accumulation of ST6Gal-I glycosyltransferase, imparting cancer stem cell characteristics in ovarian and pancreatic cancer cells^26^. Additionally, abnormal activation of the c-Met-SOX2 axis, mediated by sialylated IgG, enhances the stemness of lung cancer cells^27^. The co-regulation of LINC00857, LINC00968, LINC00663, and ITGA9-AS1 on SOX2 suggested their potential contributions in aberrant sialylation process during tumor progression.

### Two subgroups clustered by expression levels of the four sialylation-related lncRNAs

**Figure 5:**
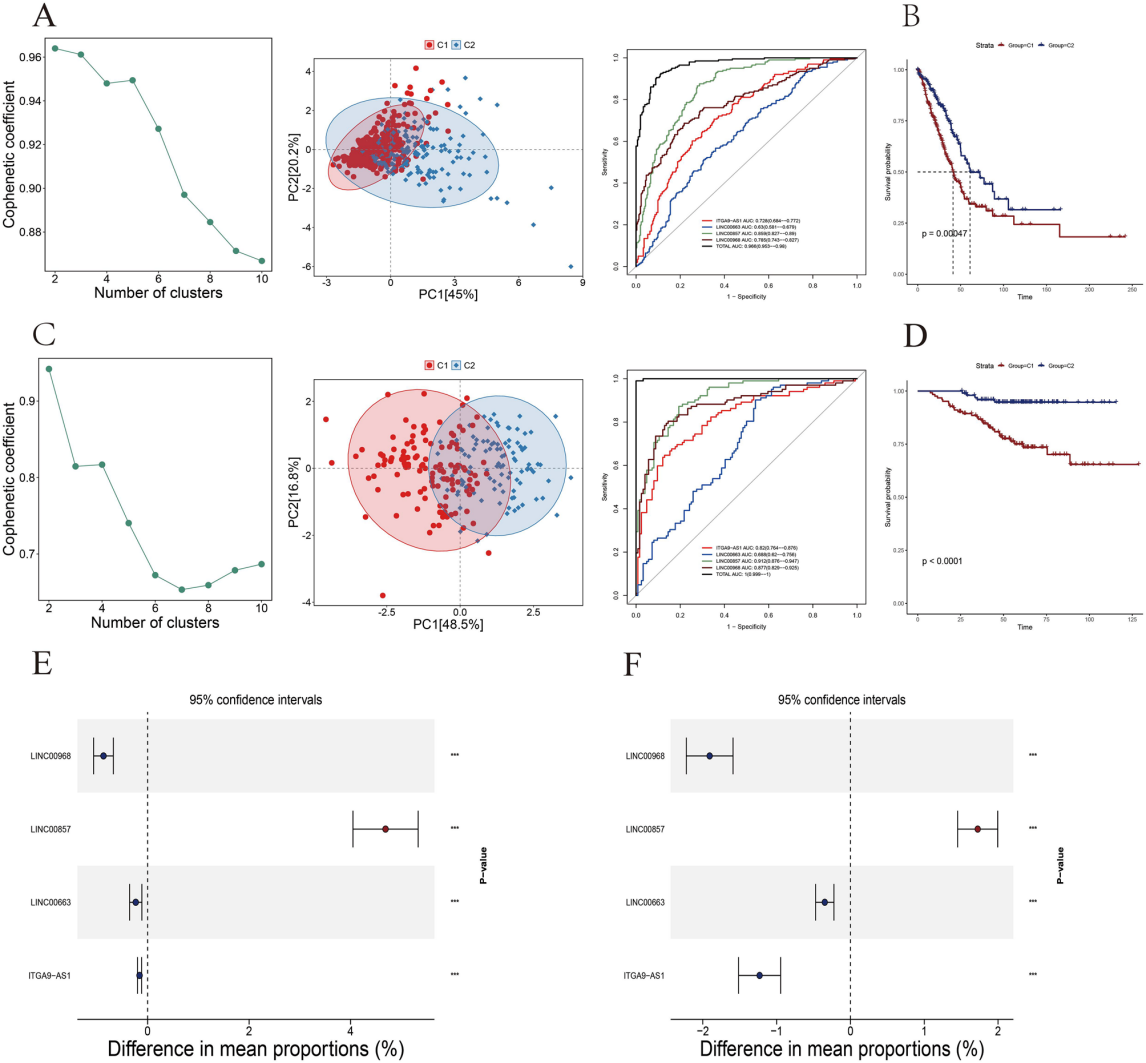
Construction of the prognostic model based on signatures of sialylation-related lncRNAs. NMF analyses grouped patients into two clusters with high stratifying performance demonstrated by ROC curves in (**A**) TCGA-LUAD and (**C**) GSE31210 data. Kaplan–Meier survival curves based on (**B**) TCGA-LUAD and (**D**) GSE31210 datasets showed significantly different clinical outcomes between C1 and C2. Difference in expression levels of four lncRNAs in (**E**) TCGA-LUAD and (**F**) GSE31210. *P < 0.05, **P < 0.01, ***P < 0.001.

To explore the performance of four sialylation-related lncRNAs in distinguishing samples with different signatures, we conducted a non-negative matrix factorization (NMF) clustering analysis. The optimum number of clusters was determined to be two in both the TCGA-LUAD (Figs. 5A, S1) and GSE31210 datasets (Figs. 5C, S1). For TCGA-LUAD data, 303 patients classified into Cluster 1 (C1), and 201 patients belonged to Cluster 2 (C2). The signature of four lncRNAs exhibited the most powerful classification ability (Total AUC: 0.966, CI: 0.953–0.98), followed by single LINC00857 (AUC: 0.859, CI: 0.827–0.89), LINC00968 (AUC: 0.785, CI: 0.743–0.827), ITGA9-AS1 (AUC: 0.728, CI: 0.684–0.772), and LINC00663 (AUC: 0.63, CI: 0.581–0.679) (Fig. 5A). Consistently, similar results were observed in GSE31210 data, with the four lncRNAs showing the highest classification ability (Total AUC: 1, CI: 0.999–1), followed by LINC00857 (AUC: 0.912, CI: 0.876–0.947), LINC00968 (AUC: 0.877, CI: 0.829–0.925), ITGA9-AS1 (AUC: 0.82, CI: 0.764–0.876), and LINC00663 (AUC: 0.688, CI: 0.62–0.756) (Fig. 5C). These results emphasized the robustness of this classification model. Survival analysis revealed significantly worse clinical outcomes for patients in C1 group compared to the C2 group in both TCGA-LUAD and GSE31210 datasets (*P* < 0.01) (Fig. 5B,D), suggesting the prognostic value of sialylation-related lncRNAs that we identified. Taken together, these findings indicated that the signature of four sialylation-related lncRNAs may contribute to stratifying patients with differential prognoses and can serve as a reliable classifier. Furthermore, unlike the other three lncRNAs, univariate COX regression analysis performed on two independent cohorts consistently uncovered that LINC00857 was significantly upregulated in C1 group rather than C2 group, indicating its potential pro-tumor role in tumor progression (Fig. 5E,F).

### Genetic alternations and underlying mechanisms associated with tumorigenesis

**Figure 6:**
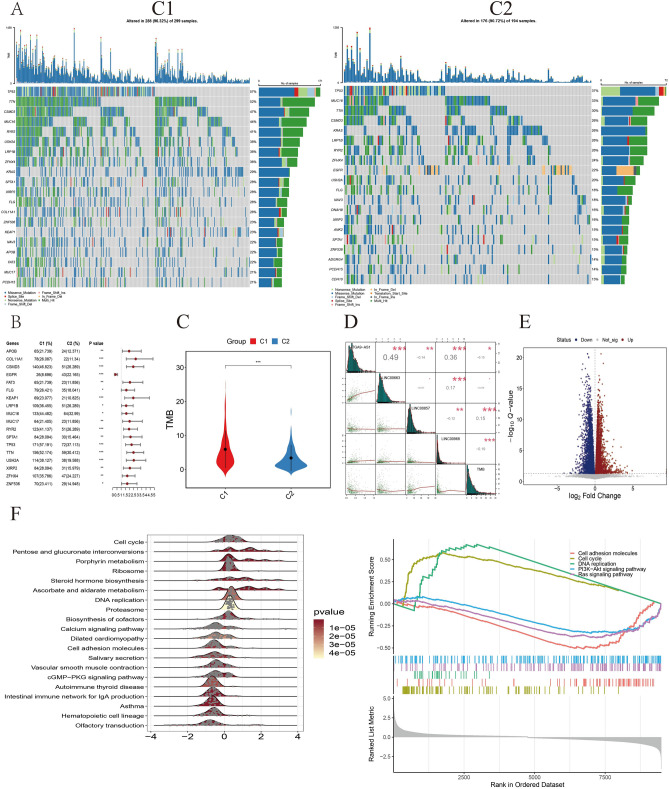
Genetic landscapes of patients in two subgroups. (**A**) Mutation landscapes of Top 20 genes ranked by mutation frequencies in C1 and C2 samples, respectively. (**B**) Genes with significantly different mutation frequencies in two clusters. (**C**) Violin plot of overall TMB scores in C1 and C2. (**D**) Correlations between TMB scores and the expression levels of four lncRNAs. (**E**) Volcano plot of DEGs between C1 and C2. (**F**) Functional enrichment analyses of DEGs. *P < 0.05, **P < 0.01, ***P < 0.001.

A total of 493 tumor samples with genomic alternations were obtained from TCGA-LUAD, with 299 samples in C1 group and 194 samples in C2 group. Although mutations of *TP53*, *TTN*, *CSMD3*, and *MUC16* predominated in both groups, higher frequencies of these mutations were detected in C1 (Fig. 6A). Among genes with significantly different mutation frequencies in two groups, most of them exhibited increased mutation frequencies in C1 group (Fig. 6B), indicating a higher TMB in C1 samples. Subsequently, all detected mutations were employed to calculate TMB scores for samples in C1 and C2 groups, respectively. Significantly higher TMB scores were revealed in C1 compared to C2 group (Fig. 6C). Furthermore, the TMB level exhibited a significantly positive correlation with the expression level of LINC00857 but was negatively related to expression levels of LINC00968, LINC00663, and ITGA9-AS1 (*P* < 0.05) (Fig. 6D). Finally, differentially expressed genes (DEGs) between C1 and C2 were recognized, and functional analyses revealed that several important cancer-related pathways were significantly enriched, such as cell cycle and DNA replication (Fig. 6E,F).

### Characterizations of tumor microenvironment in C1 and C2 groups

**Figure 7:**
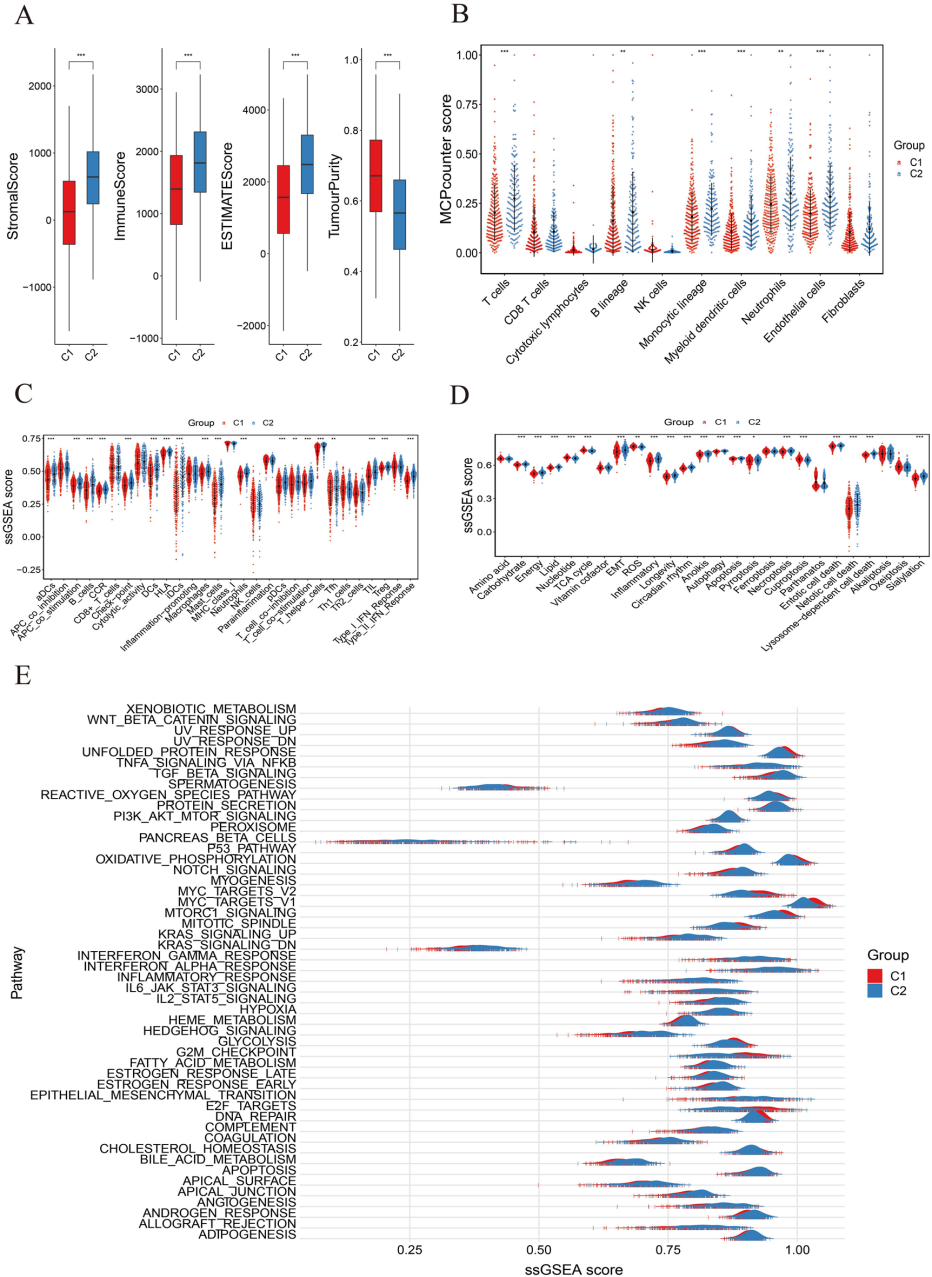
Differences of immunoinfiltration levels and pathways activities in TCGA-LUAD samples. (**A**) Box plots of TumourPurity score, StromalScore, ImmuneScore, and ESIMATEScore in C1 and C2. (**B**) MCPcounter scores and (**C**) ssGSEA scores were calculated to characterize the infiltrating levels of immune cells and related processes. ssGSEA scores of (**D**) metabolism-related pathways and (**E**) classical hallmark pathways of cancers in two subgroups. *P < 0.05, **P < 0.01, ***P < 0.001.

The tumor immune microenvironment (TIME) can be characterized by ESTIMATE algorithm at bulk transcriptome level. ImmuneScore and StromalScore were calculated to assess the proportions of infiltrating immune and stromal cells, respectively. ESTIMATEScore, consisting of ImmuneScore and StromalScore, is negatively associated with tumor purity in tumor tissues. In TCGA-LUAD dataset, significantly higher TumourPurity score was observed in C1 group, along with lower StromalScore, ImmuneScore, and ESIMATEScore (Fig. 7A). It seemed that the tumor microenviroment of C1 was characterized by low anti-tumor immunoinfiltrating levels, which may contribute to the poor survival status observed in patients of C1 group. Subsequently, significantly lower MCPcounter scores were observed for T cells, B cells, monocytic cells, myeloid dendritic cells, endothelial cells and neutrophils in C1 (*P* < 0.01) (Fig. 7B). Single Sample Gene Set Enrichment Analysis (ssGSEA) score revealed that samples in C1 group had lower scores in pathways of APC co-stimulation, B cells, CCR, Checkpoint, aDCs, DCs, iDCs, pDCs, Cytolytic activity, HLA, Macrophages, Mast cells, Neutrophils, T cell co-stimulation, T helper cells and type I/II IFN response (*P* < 0.01) (Fig. 7C). In the GSE31210 cohort, although C1 group exhibited higher ImmuneScore compared with C2, the difference was not statistically significant. However, significant differences were observed in StromalScore, ESTIMATEScore, and TumorPurity scores between the two groups (*P* < 0.01) (Fig. S2A). Besides, lower infiltrating levels of myeloid dendritic cells and endothelial cells, as well as lower ssGSEA scores of aDCs, mast cells, neutrophils, and type II IFN response were also obtained in C1 group (*P* < 0.01) (Figs. S2B,2C). In line with what we observed in TCGA-LUAD dataset, the analysis of immunoinfiltration performed on GSE31210 revealed that C1 group was characterized by relatively impaired anti-tumor immune response within tumor microenvironment.

Abnormal metabolic alternations disrupt cellular homeostasis and serve as hallmarks of cancer. Metabolic reprogramming and other abnormal signaling usually occur to adapt the requirements for survival during tumor progression. Therefore, the activities of metabolic and cancer-associated pathways were evaluated using ssGSEA analysis in the two groups. Extensive metabolic differences were observed between C1 and C2 groups. In TCGA-LUAD samples, C1 group displayed reduced Carbohydrate, Energy, Lipid, Vitamin cofactor, EMT, Longevity, Inflammatory, Circadian rhythm, Anoikis, Autophagy, Apoptosis, Pyroptosis, Necroptosis, Entotic cell death, Netotic cell death, and Lysosome-dependent cell death pathways, but Nucleotide, ROS, and Cuproptosis pathways were significantly downregulated in C2 compared with C1 group (*P* < 0.01) (Fig. 7D). Although EMT pathway was upregulated in C2, the anoikis activity was also enhanced to eliminate extracellular matrix-detached cells, suggesting the potential of inhibiting tumor metastasis. In addition, the increased activities of Energy, Longevity, and Circadian rhythm processes in C2 tumor microenvironment were validated in GSE31210 dataset, as well as the reduction of Nucleotide and ROS pathways (Fig. S2D). Notably, the Longevity pathway was significantly upregulated in C2 patients with prolonged survival time in two datasets (Fig. 7D, Fig. S2D). Further, the analysis of hallmark pathways in both TCGA-LUAD and GSE31210 cohorts revealed higher scores of glycolysis and oncogenic signaling processes in C1, including MYC targets V2, MYC targets V1, and MTORC1 pathways (Fig. 7E, Fig. S2E).

### Drug sensitivity analysis

**Figure 8:**
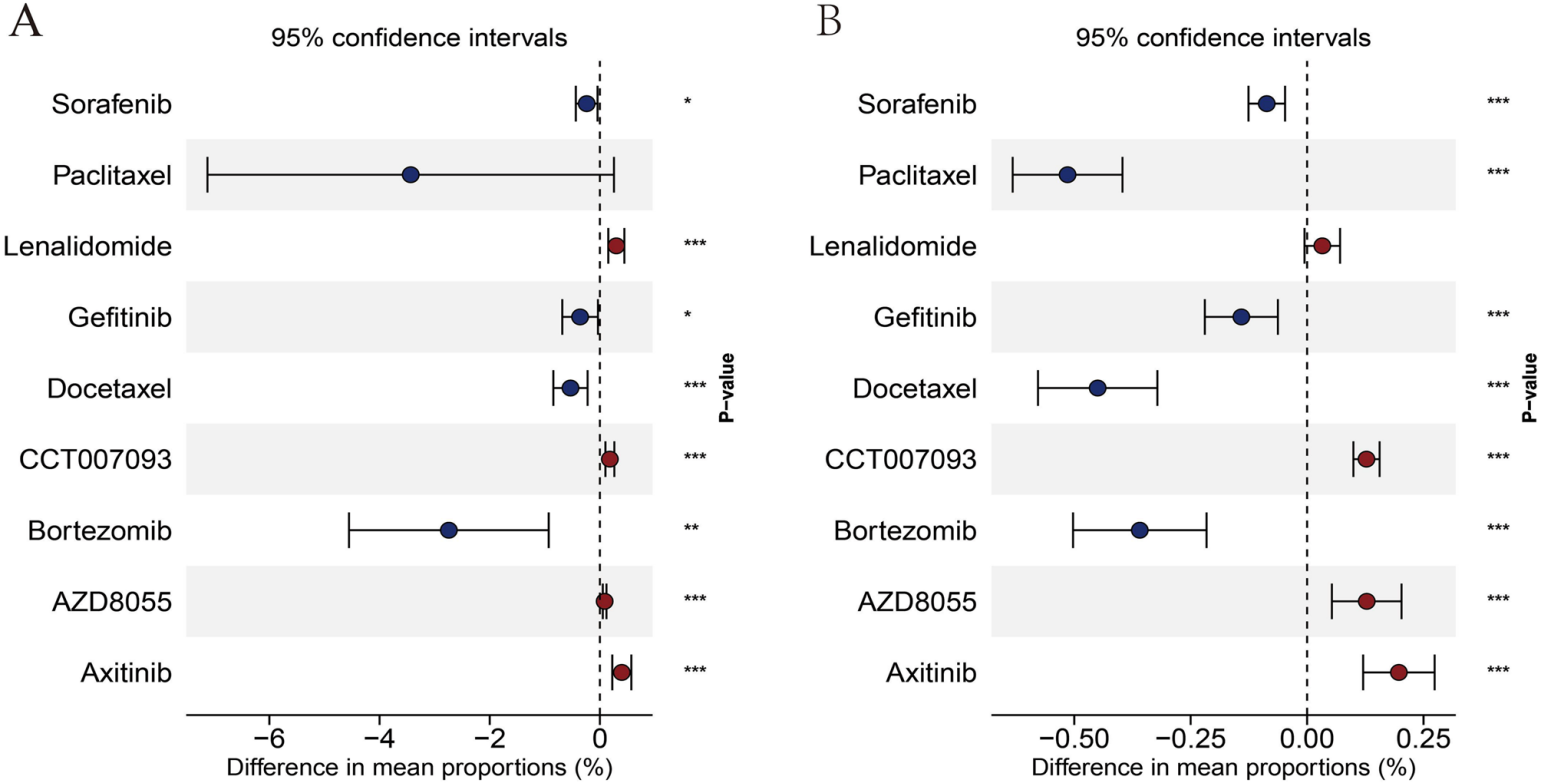
Predictions of drug responses. Significantly different IC50 of drugs in (**A**) TCGA-LUAD and (**B**) GSE31210 data. Horizontal coordinate represents the difference obtained by subtracting the median IC50 score of C2 from the median IC50 score of C1.

Tumors developed various mechanisms to resist drugs, including aberrant sialylation. To investigate the associations between sialylation levels and drug efficacy in NSCLC, drug sensitivity analysis was performed. In this study, a total of 31 drugs were included, and drugs with significantly different IC50 scores between C1 and C2 groups were identified. In both TCGA-LUAD (Fig. 8A) and GSE31210 (Fig. 8B) samples, results showed that C1 group was more sensitive to Sorafenib, Gefitinib, Docetaxel and Bortezomib, while C2 group exhibited positive responses to CCT007093, AZD8055 and Axitinib at lower concentrations, with statistical significance (*P* < 0.05). By considering the drug sensitivity analysis results, clinicians can make informed decisions regarding the selection of appropriate drugs for individual patients based on their tumor sialylation profiles. This personalized approach may enhance the efficacy of treatment and improve patient outcomes in NSCLC.

## Discussion

Aberrant epigenetic modifications are widely involved in various biological processes, promoting tumor development, invasion and metastasis. Previous studies have demonstrated the prognostic value of sialylation-related lncRNAs in CRC^25^. In this study, we identified four sialylation-related lncRNAs, namely LINC00857, LINC00663, LINC00968, and ITGA9-AS1. Results of risk analysis suggested that LINC00968, LINC00663, and ITGA9-AS1 have the potential to serve as anti-tumor regulators in LUAD, conversely, LINC00857 was identified as a risk factor. LINC00857 has been extensively associated with tumor invasion^28^, angiogenesis^29^, glycolysis^30^, immune infiltrating^31,32^, apoptosis, and autophagy^33^, and its prognostic predictive performance has been widely demonstrated in solid tumors. The overexpression of LINC00857 reduced the survival time of patients through controlling miR-1179/SPAG5 axis^30^, and its oncogenic roles have been revealed in colorectal and pancreatic cancer cells^34,35^. In this study, we found that LINC00857 regulated sialulation-related genes GALNT3, ST6GALNAC3, CHST1, and GALNT7 by targeting miR-486-5p/ST6GALNAC6, miR-150-5P/CMAS, and miR-340-5p. It has been reported that LINC00968 inhibits the proliferation of lung cancer cells by targeting miR-9-5p/CPEB3 and miR-21-5p/SMAD7 and exhibits downregulation in tumors compared to normal tissues^36^. The mechanisms by which LINC00663 regulates tumorigenesis remain unclear. Although LINC00663 can suppress the expression of oncogene ENO1 in breast cancer^37^, it was upregulated in pancreatic cancer and glioma cells, promoting tumor progression^38,39^. The roles of ITGA9-AS1 and the interactions among the four lncRNAs have been relatively less studied. Notably, we observed their interactions with three genes, namely IGF2BP3, SOX2, and IGFBP1. Although the potential regulatory mechanisms of IGF2BP3 and IGF2BP1 require further exploration, it has been shown that SOX2 regulates the stemness of cancer cells by mediating sialylation processes, emphasizing the significance of the four lncRNAs in sialylation and tumor progression^26,27^.

Based on the expression levels of the four sialylation-related lncRNAs, two clusters with different characterizations were recognized. C1 exhibited the upregulation of pro-tumor LINC00857, while anti-tumor lncRNAs LINC00968, LINC00663, and ITGA9-AS1 were upregulated in C2. Genomic analysis revealed a higher tumor mutation burden in C1, and subsequent survival curves uncovered that patients in C1 group had significantly shorter overall survival (OS) compared with C2 group. Further explorations focused on tumor microenvironment were carried out to identify the potential contributors of different prognoses between the two subgroups. Our analyses performed on TCGA-LUAD and GSE31210 indicated that the immune microenvironment of C2 was characterized by the upregulated aDCs, mast cells, and neutrophils. Previous studies have reported the anti-tumor activity and promoting tumor immunogenicity of IFNγ^40^, and we predicted a more activated Type_II_IFN response in C2 group. The tumor suppressor, APC, which is involved in multiple pathways^41^, was significantly upregulated in C2. LncRNAs can mediate the PTM of proteins in multiple metabolic pathways, such as metabolic enzymes and transcription factors^42^. It was reported that upregulated LINC00857 alternated lipid metabolism in LUAD^43^. Consistently, we observed different activities of lipid pathway between the two groups based on TCGA-LUAD samples. In addition, C2 groups exhibited higher scores of Longevity and Circadian rhythm in both GSE31210 and TCGA-LUAD datasets. In studies of head and neck squamous cell carcinoma, circadian rhythm-related genes have been identified as independent risk factors^44^. The roles of ROS in tumor progression, including promoting DNA damage, EMT, and the formation of immunosuppressive microenvironment, have been previously reviewed^45^. Samples in C1 group accumulated higher levels of ROS, in line with their survival disadvantages. Furthermore, C1 group showed higher ssGSEA scores of DNA repair, MYC targets V1/V2, and MTORC1 signaling pathways in the two cohorts. These pathways are associated with cell proliferation and are positively related to tumor malignancy in pancancer^46,47^. As one of the hallmarks of cancer, the higher score for cell cycle pathway in C1 indicated stronger proliferation abilities of lung cancer cells. Upregulated glycolysis was also observed in C1, facilitating to meet the energy needs of cell proliferation^48^. Taken together, our findings suggested the existence of two distinct groups characterized by different clinical outcomes, immune profiles, and metabolic microenvironment in LUAD.

The association between sialylation with therapeutic efficacy has been revealed in cancer. For example, targeting ST6GALNAC2 has been shown to reverse the resistance of CRC cells to 5-fluorouracil^49^. While the correlations and regulatory mechanisms related to sialylation in NSCLC have been relatively understudied. In this study, drug sensitivity analysis was carried out, and we identified several drugs that exhibited different effects. Compared to C2, patients in C1 seemed to benefit from Bortezomib, Docetaxel, Gefitinib, Paclitaxel, and Soragenib, which can be supported by previous studies. For example, the pan-sialyltransferase inhibitor 3Fax-Neu5Ac has been shown to enhance the therapeutic efficacy of Bortezomib in mouse models of multiple myeloma, suggesting the reduced level of sialylation is positively associated with Bortezomib sensitivity^50^. Consistently, pathway analysis of TCGA-LUAD samples revealed lower sialylation scores in C1, and higher Bortezomib sensitivity was also observed. Overexpressed ST6Gal-I has been reported to upregulate α2,6-sialylation of FGFR1, inducing Paclitaxel resistance in ovarian cancer^51^, in line with the higher sialylation level and lower Paclitaxel sensitivity in C2. Similarly, ST6Gal-1 promotes the sialylation of EGFR, which further weakens the Gefitinib effectiveness in CRC^52,53^. Here, we also found that C2, with higher sialylation levels, exhibited increased half-maximal inhibitory concentration (IC50). No studies have investigated the roles of sialylation in Sorafenib, Axitinib, AZD8055, CCT007093, and Lenalidomide therapies. Our results suggested the potential application of Axitinib, AZD8055, CCT007093, Lenalidomide in C2, and Sorafenib in C1. These findings have important clinical implications as they could potentially facilitate the development of personalized therapy strategies based on the sialylation status of patients.

Given the deficiency of large cohorts with clinical samples, the stratification ability of our prognostic model requires further validation. Regarding the existence of the four lncRNAs in clinical samples, we have only confirmed their differential expression profiles between tumor and adjacent tissues in four patients. However, based on our findings, it would be ideal to validate these results in samples specifically classified as C1 and C2. Furthermore, the lncRNA-mediated regulatory network was predicted using bioinformatics methods, and there is a lack of in vivo or in vitro experiments to confirm the actual synergistic changes. In other words, this regulatory network has not been validated. Additionally, based on bulk transcriptomic data, we have only characterized the overall differences in pathways and immunoinfiltrating levels between C1 and C2. However, we have not investigated the underlying reasons for these differences or explored the interconnectedness of these distinct biological processes. Further research is needed to delve into these aspects, such as employing a more refined resolution at the single-cell level to investigate the differences in the dynamic tumor immune microenvironment between the groups. Finally, our findings just serve as a foundation for identifying potential new biomarkers or therapeutic targets, however, further research investments will be necessary for the development of drugs in the future.

## Conclusion

We demonstrated the clustering performance of four sialylation-related lncRNAs, which successfully classified LUAD patients into two groups characterized by different signatures in tumor mutation burden, immune infiltration, metabolic alternation, drug sensitivity, and clinical outcome. Our findings supported the potential roles of four lncRNAs in sialylation, and suggested the prognostic value of lncRNA, providing some insights into precise treatment.

## Materials and methods

### Data source

A total of 504 patients with clinical data of LUAD were enrolled for analysis, and the expression data of tumor tissues plus adjacent samples were retrospectively collected from TCGA (https://cancergenome.nih.gov/). Somatic mutation data were also downloaded from TCGA database. At the same time, GSE31210 (N = 226) dataset from the Gene Expression Omnibus (GEO) database (https://www.ncbi.nlm.nih.gov/geo/) was applied as a validated cohort.

### Identification of sialylation-related lncRNA associated with patient prognosis

To identify genes potentially involved in sialylation process, pathways associated with sialyltransferases, transporters, and neuraminidases were collected from the Molecular Signatures Database (MSigDB) (https://www.gsea-msigdb.org/gsea/msigdb) (Table S1)^54,55^. With the TPM matrix data of samples, we independently performed Pearson correlation analysis on TCGA-LUAD and GSE31210 datasets using a self-developed function to roughly recognize sialylation-related lncRNAs (Tables S2, S3). The filtering criteria used were the significance level of *P* ≤ 0.05 and an absolute value of correlation coefficient > 0.3. LncRNAs that meet these criteria were considered sialylation-related, and 70 lncRNAs shared by both TCGA-LUAD and GSE31210 were left for downstream analysis. Using the survival package (v3.5.5), and then, the univariate Cox proportional regression analysis was respectively carried out in training set and test set to screen sialylation-related lncRNAs associated with survival time, and 5 lncRNAs (LINC00857, LINC00663, LINC00968, ITGA9-AS1, TBX5-AS1) with statistical significance and shared by two datasets were kept. At the same time, the hazard ratio of each lncRNA was obtained. With transcriptomic data of the 5 lncRNAs as input for TCGA-LUAD samples, we employed ‘rfsrc’ function of randomForestSRC package (v3.2.3) to construct a random survival forest model (N_trees_ = 1000) and obtained the variable importance value for each lncRNA. After ranking all output lncRNAs, four lncRNAs with variable importance exceeding 0.02 were included for downstream analyses (N_lncRNA_ = 4; ITGA9-AS1, LINC00663, LINC00857, LINC00968). Finally, for both TCGA-LUAD and GSE31210 datasets, multivariate COX proportional regression analysis was separately employed to evaluate the risk score for each patient. Specifically, risk score = 1.048 * ${\text{exp}}_{{{\text{ITGA}}9 - {\text{AS}}1}}$ + 0.794 * ${\text{exp}}_{{{\text{LINC}}00663}}$ + 1.050 * ${\text{exp}}_{{{\text{LINC}}00857}}$ + 0.752 * $\exp_{{{\text{LINC00968}}}}$. The threshold value dividing patients into high-risk group and low-risk group was determined by ‘surv_cutpoint’ function of survminer package (v0.4.9). Kaplan–Meier survival curves were profiled between high- and low-risk groups utilizing survival and survminer packages (v0.4.9) in R.

### Predicted regulatory network regulated by four sialylation-related lncRNA

Based on the databases of lnc2cancer (http://www.bio-bigdata.net/lnc2cancer/), lncRNAdisease (http://www.cuilab.cn/lncrnadisease), and RNADisease (http://www.rnadisease.org/), the known associations between sialylation-related RNAs (lncRNA and mRNA) and cancers were collected and visualized by disease enrichment analysis with in-house method. The known lncRNA-miRNA interaction pairs were obtained from databases of starbase (http://starbase.sysu.edu.cn/index.php) and NPInter5 (http://bigdata.ibp.ac.cn/npinter5/), and bedtools package (v2.26.0) was utilized to predict respective target genes of the four core lncRNAs. Regulatory network among sialylation-related molecules was visualized utilizing cytoscape package (v3.8.2). Pathways consisting of targeted genes were collected from Kyoto Encyclopedia of Genes and Genomes (https://www.kegg.jp/), and ccgraph package (https://github.com/gaospecial/ccgraph) was utilized to plot the dendrogram that illustrates the relationships between genes and pathways.

### RT-qPCR

Four patients clinically diagnosed with LUAD were enrolled in this study. Paired tumor and normal tissues were sampled and placed in a mortar for cryogenic grinding with liquid nitrogen. The ground tissue powder was then transferred to a sterile, enzyme-free 1.5 ml tube, and total RNA was extracted using TRIzol reagent. Concentration and quality of total RNA were determined using an enzyme labeling instrument, and the PrimeScript RT kit (Takara, Dalian, China) was used for reverse transcription of mRNA. Synthesized cDNA, together with SYBR Premix Ex TaqTM II (Tli RNaseH Plus) (Takara) and primers were added to a 96-well PCR plate in a Bio-Rad CFX 96 real-time PCR system (Bio-Rad Laboratories, Inc., Hercules, CA, USA). The Primer Blast tool (https: //www.ncbi.nlm.nih.gov/tools/primer-blast/) was utilized for online primer design, with following primers: GAPDH (Forward: 5ʹ-CCAGCAAGAGCACAAGAGGA-3ʹ. Reverse: 5ʹ-TGAGGAGGGGAGATTCAGTGT-3ʹ), LINC00857 (Forward: 5ʹ-TGAGACATGTTGCAGACCCC-3ʹ. Reverse: 5ʹ-TCTTCTTGCGCTTCGTCAGT-3ʹ), LINC00968 (Forward: 5ʹ-CACCCACTGGTCCATTTGGA-3ʹ. Reverse: 5ʹ-TGTGCTGAGCTGTCTGGAAG-3’). LINC00663 (Forward: 5ʹ-CATTGATCGCCTGACCTCCA-3ʹ. Reverse: 5ʹ-AGCCTCTGGGTGACACATTG-3ʹ). ITGA9-AS1 (Forward: 5ʹ-TCCATGCCAGGTCTGTTCTG-3ʹ Reverse: 5ʹ-GAGCCAGACAGCTTATGGGA-3ʹ). Among these, GAPDH was an internal reference gene. The relative gene expressions were calculated using the 2^−ΔΔCT^ method. *P* value < 0.05 was considered statistically significant.

### Construction and validation of a prognostic model

With the transcriptomic data (TPM matrix) of four lncRNAs, the NMF clustering was performed by the NMF package (v0.26) in R. The optimal dimension was determined based on the point at which the cophenetic correlation coefficient initially decreased^56^. Based on the cluster category label obtained from NMF clustering, the sialylation-related lncRNA matrix was utilized for PCA dimensionality reduction. Additionally, we established logistic regression relationships based on the signatures of the labels of C1 plus C2 and lncRNAs, to plot the receiver operating characteristic (ROC) curves. Finally, the Kaplan–Meier survival analysis was carried out to characterize the different clinical signatures of C1 and C2.

### Characterization of genetic alternation

Based on somatic mutation data for LUAD obtained from TCGA, we profiled the separate mutation landscapes in C1 and C2 groups by the maftools package. Differential mutation frequencies were compared between the two subtypes using the Fisher-test. For samples in two groups, ssGSEA scores of hallmark pathways collected from MSigDB database were calculated by GSVA package (v1.48.2) in R, and visualization of results was achieved by in-house developed programs.

### Signatures of tumor microenvironment in C1 and C2 groups

Tumor immune microenvironment (TIME) at the bulk level is usually evaluated using ESTIMATE (Estimation of STromal and Immune cells in MAlignant Tumor tissues using Expression data) algorithm (v1.0.13). This algorithm primarily utilizes ssGSEA for gene expression data analysis, generating ImmuneScore, ESTIMATEScore, and StromalScore. ImmuneScore and StromalScore are utilized to indicate the inferred infiltration levels of immune cells and stromal cells. ESTIMATEScore, consisting of ImmuneScore and StromalScore, is utilized to estimate tumor purity in tumor tissues^57,58^. Subsequently, the ssGSEA algorithm was employed to elucidate the enrichment of 29 immune-related gene sets, and MCPcounter (https://github.com/ebecht/MCPcounter) was applied to quantify the relative proportions of infiltrating immune cells, aiming to assess the composition and abundance of immune cells within tumor microenvironment. In addition, considering the dysregulated metabolic microenvironment during tumor progression, metabolic-related pathways were collected from MSigDB database, and the ssGSEA scores were calculated in the two groups using GSVA package (v1.48.2).

### Prediction of response to drug therapy

IC50 is usually utilized to evaluate the effectiveness of drugs in inhibiting specific biological or biochemical functions in preclinical studies. The R package of pRRophetic (v0.5) was employed to predict IC50 of common chemotherapeutic agents. The difference between the two groups was tested by the Wilcoxon signed-rank test.

### Statistical analysis

R software (version 4.3.1) was utilized for data processing and visualization. Continuous variables were analyzed using the Wilcoxon rank-sum test or Student’s t-test, and categorical variables were analyzed by Fisher’s exact test. Pearson correlation and Spearman’s correlation analyses were employed to assess the relationships between mRNA and lncRNA pairs. To evaluate the significance of differences in Kaplan–Meier survival curves between groups, the log-rank test was used. *P* value < 0.05 was considered statistically significant.

### Supplementary Information

Supplementary Information 1.

Supplementary Information 2.

Supplementary Information 3.

Supplementary Information 4.

Supplementary Information 5.

Supplementary Information 6.

## Data Availability

All data generated or analyzed during this study are included in this article.

## References

[1] Pikor LA, Ramnarine VR, Lam S, Lam WL (2013). Genetic alterations defining NSCLC subtypes and their therapeutic implications. Lung Cancer..

[2] Gulhane P, Singh S (2023). Unraveling the Post-Translational Modifications and therapeutical approach in NSCLC pathogenesis. Trans. Oncol..

[3] Varki A, Kornfeld S, Varki A, Cummings RD, Esko JD, Stanley P, Hart GW, Aebi M (2022). Historical background and overview. Essentials of Glycobiology.

[4] Munkley J (2022). Aberrant sialylation in cancer: Therapeutic opportunities. Cancers..

[5] Yakubu RR, Nieves E, Weiss LM (2019). The methods employed in mass spectrometric analysis of posttranslational modifications (PTMs) and protein-protein interactions (PPIs). Adv. Exp. Med. Biol..

[6] Harduin-Lepers A, Vallejo-Ruiz V, Krzewinski-Recchi MA, Samyn-Petit B, Julien S, Delannoy P (2001). The human sialyltransferase family. Biochimie.

[7] Schjoldager KT, Narimatsu Y, Joshi HJ, Clausen H (2020). Global view of human protein glycosylation pathways and functions. Nat. Rev. Mol. Cell. Biol..

[8] Dobie C, Skropeta D (2021). Insights into the role of sialylation in cancer progression and metastasis. Br. J. Cancer..

[9] Pietrobono S, Stecca B (2021). Aberrant sialylation in cancer: Biomarker and potential target for therapeutic intervention?. Cancers (Basel)..

[10] Yuan Y, Wu L, Shen S, Wu S, Burdick MM (2016). Effect of alpha 2,6 sialylation on integrin-mediated adhesion of breast cancer cells to fibronectin and collagen IV. Life Sci..

[11] Seales EC, Jurado GA, Brunson BA, Wakefield JK, Frost AR, Bellis SL (2005). Hypersialylation of beta1 integrins, observed in colon adenocarcinoma, may contribute to cancer progression by up-regulating cell motility. Cancer Res..

[12] Seales EC, Jurado GA, Singhal A, Bellis SL (2003). Ras oncogene directs expression of a differentially sialylated, functionally altered β1 integrin. Oncogene..

[13] Sakuma K, Aoki M, Kannagi R (2012). Transcription factors c-Myc and CDX2 mediate E-selectin ligand expression in colon cancer cells undergoing EGF/bFGF-induced epithelial-mesenchymal transition. Proc. Natl. Acad. Sci. U S A..

[14] Huang J, Huang J, Zhang G (2022). Insights into the role of sialylation in cancer metastasis, immunity, and therapeutic opportunity. Cancers (Basel)..

[15] Li J, Long Y, Sun J, Wu J, He X, Wang S (2022). Comprehensive landscape of the ST3GAL family reveals the significance of ST3GAL6-AS1/ST3GAL6 axis on EGFR signaling in lung adenocarcinoma cell invasion. Front. Cell Dev. Biol..

[16] Perez S, Fu CW, Li WS (2021). Sialyltransferase inhibitors for the treatment of cancer metastasis: current challenges and future perspectives. Molecules..

[17] Gong L, Zhou X, Yang J, Jiang Y, Yang H (2017). Effects of the regulation of polysialyltransferase ST8SiaII on the invasiveness and metastasis of small cell lung cancer cells. Oncol Rep..

[18] Büll C, Boltje T, van Dinther EA, Peters T, de Graaf AM, Leusen JH, Kreutz M, Figdor CG, den Brok MH, Adema GJ (2015). Targeted delivery of a sialic acid-blocking glycomimetic to cancer cells inhibits metastatic spread. ACS Nano..

[19] Lakshmanan I, Chaudhary S, Vengoji R, Seshacharyulu P, Rachagani S, Carmicheal J (2021). ST6GalNAc-I promotes lung cancer metastasis by altering MUC5AC sialylation. Mol Oncol..

[20] Beatson R, Tajadura-Ortega V, Achkova D, Picco G, Tsourouktsoglou TD, Klausing S (2016). The mucin MUC1 modulates the tumor immunological microenvironment through engagement of the lectin Siglec-9. Nat. Immunol..

[21] Li B, Zhang B, Wang X, Zeng Z, Huang Z, Zhang L (2020). Expression signature, prognosis value, and immune characteristics of Siglec-15 identified by pan-cancer analysis. Oncoimmunology..

[22] Yuan Q, Chen X, Han Y, Lei T, Wu Q, Yu X (2018). Modification of alpha2,6-sialylation mediates the invasiveness and tumorigenicity of non-small cell lung cancer cells in vitro and in vivo via Notch1/Hes1/MMPs pathway. Int. J. Cancer..

[23] Chiang CH, Wang CH, Chang HC, More SV, Li WS, Hung WC (2010). A novel sialyltransferase inhibitor AL10 suppresses invasion and metastasis of lung cancer cells by inhibiting integrin-mediated signaling. J. Cell. Physiol..

[24] Gong A, Zhao X, Pan Y, Qi Y, Li S, Huang Y (2020). The lncRNA MEG3 mediates renal cell cancer progression by regulating ST3Gal1 transcription and EGFR sialylation. J. Cell Sci..

[25] Zhou M, Lv S, Hou Y, Zhang R, Wang W, Yan Z (2022). Characterization of sialylation-related long noncoding RNAs to develop a novel signature for predicting prognosis, immune landscape, and chemotherapy response in colorectal cancer. Front. Immunol..

[26] Dorsett KA, Jones RB, Ankenbauer KE, Hjelmeland AB, Bellis SL (2019). Sox2 promotes expression of the ST6Gal-I glycosyltransferase in ovarian cancer cells. J. Ovarian Res..

[27] Huang X, Zhang S, Tang J, Tian T, Pan Y, Wu L (2023). A self-propagating c-Met-SOX2 axis drives cancer-derived IgG signaling that promotes lung cancer cell stemness. Cancer Res..

[28] Mao Y, Cai F, Jiang T, Zhu X (2023). Identification invasion-related long non-coding RNAs in lung adenocarcinoma and analysis of competitive endogenous RNA regulatory networks. Int. J. Gen. Med..

[29] Gong Q, Huang X, Chen X, Zhang L, Zhou C, Li S (2023). Construction and validation of an angiogenesis-related lncRNA prognostic model in lung adenocarcinoma. Front. Genet..

[30] Wang L, Cao L, Wen C, Li J, Yu G, Liu C (2020). LncRNA LINC00857 regulates lung adenocarcinoma progression, apoptosis and glycolysis by targeting miR-1179/SPAG5 axis. Hum. Cell..

[31] Mu L, Ding K, Tu R, Yang W (2021). Identification of 4 immune cells and a 5-lncRNA risk signature with prognosis for early-stage lung adenocarcinoma. J. Transl. Med..

[32] Ren X, Liu J, Wang R, Liu X, Ma X, Lu Z (2022). Exploring the oncogenic roles of LINC00857 in pan-cancer. Front. Pharmacol..

[33] Su W, Wang L, Zhao H, Hu S, Zhou Y, Guo C (2020). LINC00857 interacting with YBX1 to regulate apoptosis and autophagy via MET and phosphor-AMPKa signaling. Mol. Ther. Nucleic Acids..

[34] Tang S, Liu Q, Xu M (2021). LINC00857 promotes cell proliferation and migration in colorectal cancer by interacting with YTHDC1 and stabilizing SLC7A5. Oncol. Lett..

[35] Zhang W, Qian W, Gu J, Gong M, Zhang W, Zhang S (2023). Mutant p53 driven-LINC00857, a protein scaffold between FOXM1 and deubiquitinase OTUB1, promotes the metastasis of pancreatic cancer. Cancer Lett..

[36] Zhu Y, Bo H, Chen Z (2020). LINC00968 can inhibit the progression of lung adenocarcinoma through the miR-21-5p/SMAD7 signal axis. Aging (Albany NY)..

[37] Ma J, Zhu J, Li J, Liu J, Kang X, Yu J (2023). Enhanced E6AP-mediated ubiquitination of ENO1 via LINC00663 contributes to radiosensitivity of breast cancer by regulating mitochondrial homeostasis. Cancer Lett..

[38] Ke M (2022). Identification and validation of apparent imbalanced Epi-lncRNAs prognostic model based on multi-omics data in pancreatic cancer. Front. Mol. Biosci..

[39] Pan M, Shi J, Yin S, Meng H, He C, Wang Y (2021). The effect and mechanism of LINC00663 on the biological behavior of glioma. Neurochem. Res..

[40] Dunn GP, Koebel CM, Schreiber RD (2006). Interferons, immunity and cancer immunoediting. Nat. Rev. Immunol..

[41] Abbott J, Nathke IS (2023). The adenomatous polyposis coli protein 30 years on. Semin. Cell. Dev. Biol..

[42] Tan YT, Lin JF, Li T, Li JJ, Xu RH, Ju HQ (2021). LncRNA-mediated posttranslational modifications and reprogramming of energy metabolism in cancer. Cancer Commun. (Lond)..

[43] Zhao J, Li G, Zhao G, Wang W, Shen Z, Yang Y (2022). Prognostic signature of lipid metabolism associated LncRNAs predict prognosis and treatment of lung adenocarcinoma. Front. Oncol..

[44] Aye L, Wang Z, Chen F, Xiong Y, Zhou J, Wu F (2023). Circadian regulator-mediated molecular subtypes depict the features of tumor microenvironment and indicate prognosis in head and neck squamous cell carcinoma. J. Immunol. Res..

[45] Cheung EC, Vousden KH (2022). The role of ROS in tumour development and progression. Nat. Rev. Cancer..

[46] Schulze A, Oshi M, Endo I, Takabe K (2020). MYC targets scores are associated with cancer aggressiveness and poor survival in ER-positive primary and metastatic breast cancer. Int. J. Mol. Sci..

[47] Oshi M, Gandhi S, Yan L, Tokumaru Y, Wu R, Yamada A (2022). Abundance of reactive oxygen species (ROS) is associated with tumor aggressiveness, immune response, and worse survival in breast cancer. Breast Cancer Res. Treat..

[48] Ganapathy-Kanniappan S, Geschwind JF (2013). Tumor glycolysis as a target for cancer therapy: Progress and prospects. Mol. Cancer..

[49] Liu B, Liu Y, Zhao L, Pan Y, Shan Y, Li Y (2017). Upregulation of microRNA-135b and microRNA-182 promotes chemoresistance of colorectal cancer by targeting ST6GALNAC2 via PI3K/AKT pathway. Mol. Carcinog..

[50] Natoni A, Farrell ML, Harris S, Falank C, Kirkham-McCarthy L, Macauley MS (2020). Sialyltransferase inhibition leads to inhibition of tumor cell interactions with E-selectin, VCAM1, and MADCAM1, and improves survival in a human multiple myeloma mouse model. Haematologica..

[51] Ou L, He X, Liu N, Song Y, Li J, Gao L (2020). Sialylation of FGFR1 by ST6Gal-I overexpression contributes to ovarian cancer cell migration and chemoresistance. Mol. Med. Rep..

[52] Britain CM, Holdbrooks AT, Anderson JC, Willey CD, Bellis SL (2018). Sialylation of EGFR by the ST6Gal-I sialyltransferase promotes EGFR activation and resistance to gefitinib-mediated cell death. J. Ovarian Res..

[53] Chang TC, Chin YT, Nana AW, Wang SH, Liao YM, Chen YR (2018). Enhancement by nano-diamino-tetrac of antiproliferative action of gefitinib on colorectal cancer cells: Mediation by EGFR sialylation and PI3K activation. Horm. Cancer..

[54] Liberzon A, Birger C, Thorvaldsdottir H, Ghandi M, Mesirov JP, Tamayo P (2015). The molecular signatures database (MSigDB) hallmark gene set collection. Cell Syst..

[55] Liberzon A, Subramanian A, Pinchback R, Thorvaldsdottir H, Tamayo P, Mesirov JP (2011). Molecular signatures database (MSigDB) 3.0. Bioinformatics..

[56] Brunet JP, Tamayo P, Golub TR, Mesirov JP (2004). Metagenes and molecular pattern discovery using matrix factorization. Proc. Natl. Acad. Sci. U S A..

[57] Yoshihara K, Shahmoradgoli M, Martinez E, Vegesna R, Kim H, Torres-Garcia W (2013). Inferring tumour purity and stromal and immune cell admixture from expression data. Nat. Commun..

[58] Aran D, Sirota M, Butte AJ (2015). Systematic pan-cancer analysis of tumour purity. Nat. Commun..

