# Supplementary figures and images for: The prognostic value of sialylation-related long non-coding RNAs in lung adenocarcinoma
Source: Sci Rep. 2024 Apr 17;14:8879. doi: 10.1038/s41598-024-59130-3 (PMC11024174; doi:10.1038/s41598-024-59130-3)

A

NMF rank survey

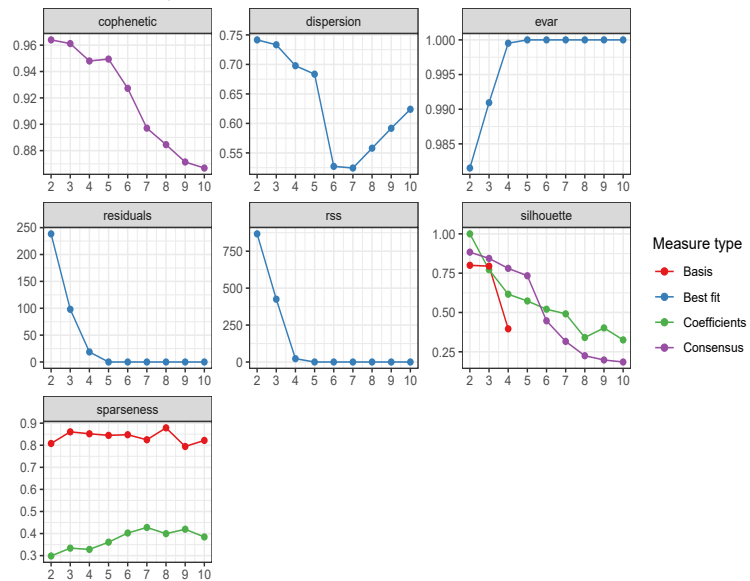

B

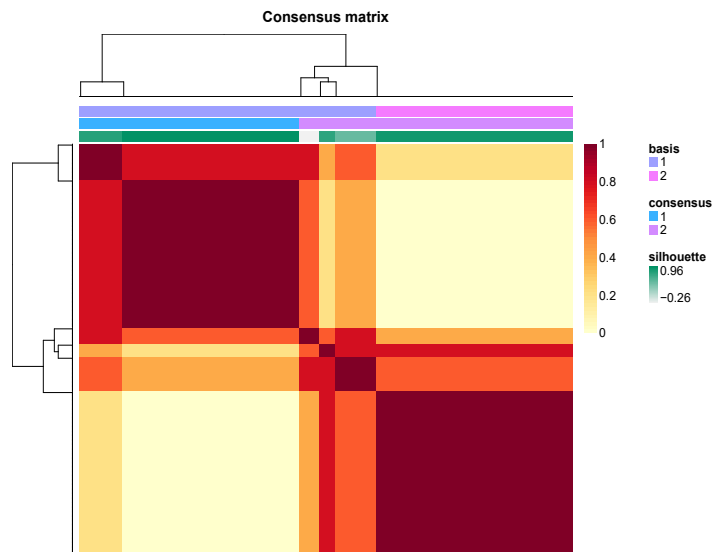

C

NMF rank survey

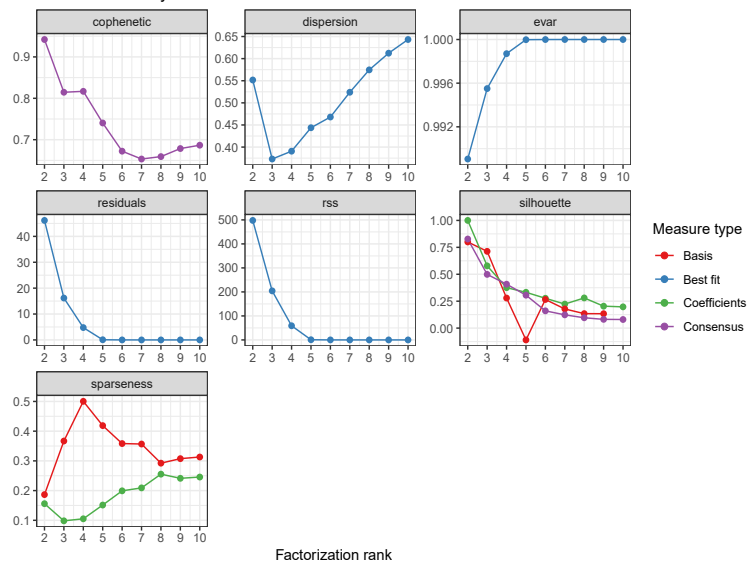

D

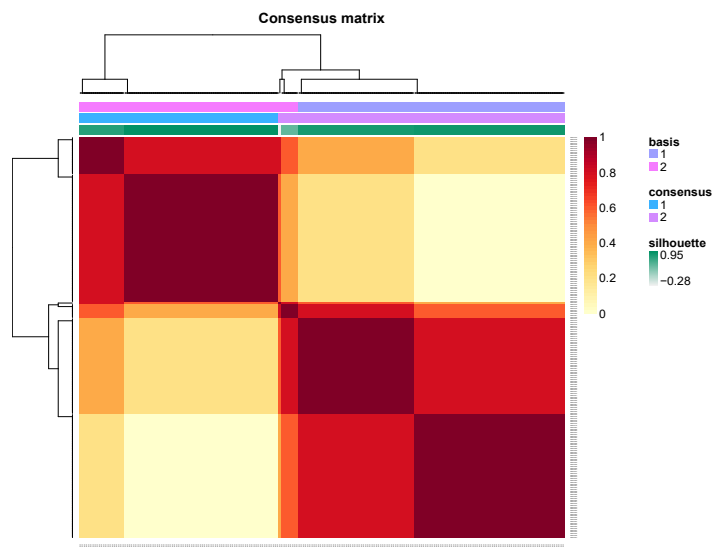

Supplement: Supplementary file 1 — Supplementary Information 1. [file 41598_2024_59130_MOESM1_ESM.pdf]
