# Supplementary material for: The prognostic value of sialylation-related long non-coding RNAs in lung adenocarcinoma
Source: Sci Rep. 2024 Apr 17;14:8879. doi: 10.1038/s41598-024-59130-3 (PMC11024174; doi:10.1038/s41598-024-59130-3)
Supplement: Supplementary file 3 — Supplementary Information 3. [file 41598_2024_59130_MOESM3_ESM.docx]

**Supplementary figures**

**
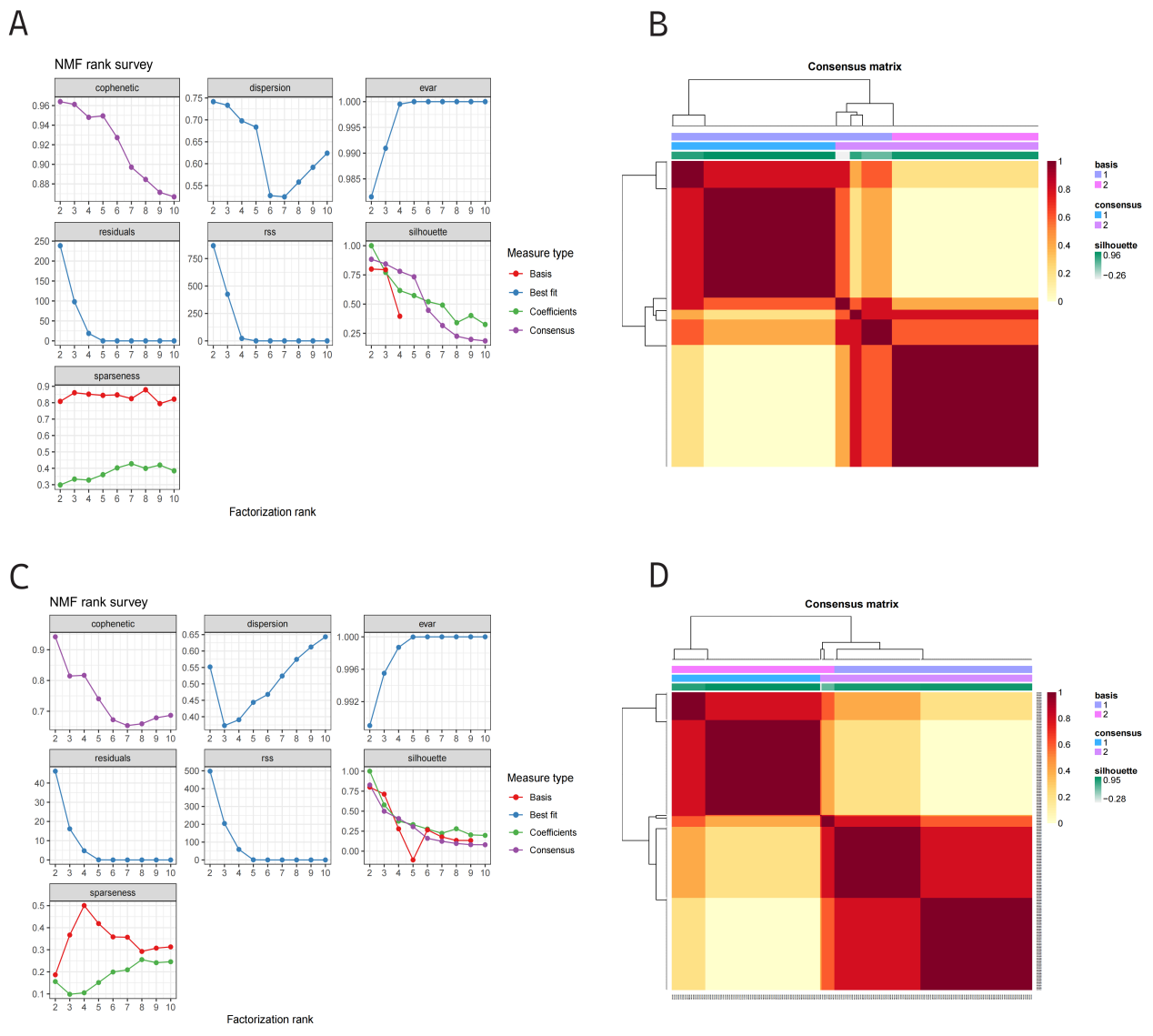
**

**Figure S1.** NMF clustering results in TCGA-LUAD and GSE31210. Estimation of the rank based on **(A)** TCGA-LUAD and **(C)** GSE31210 datasets. Consensus maps of NMF clustering in **(B)** TCGA-LUAD and **(D)** GSE31210 datasets.


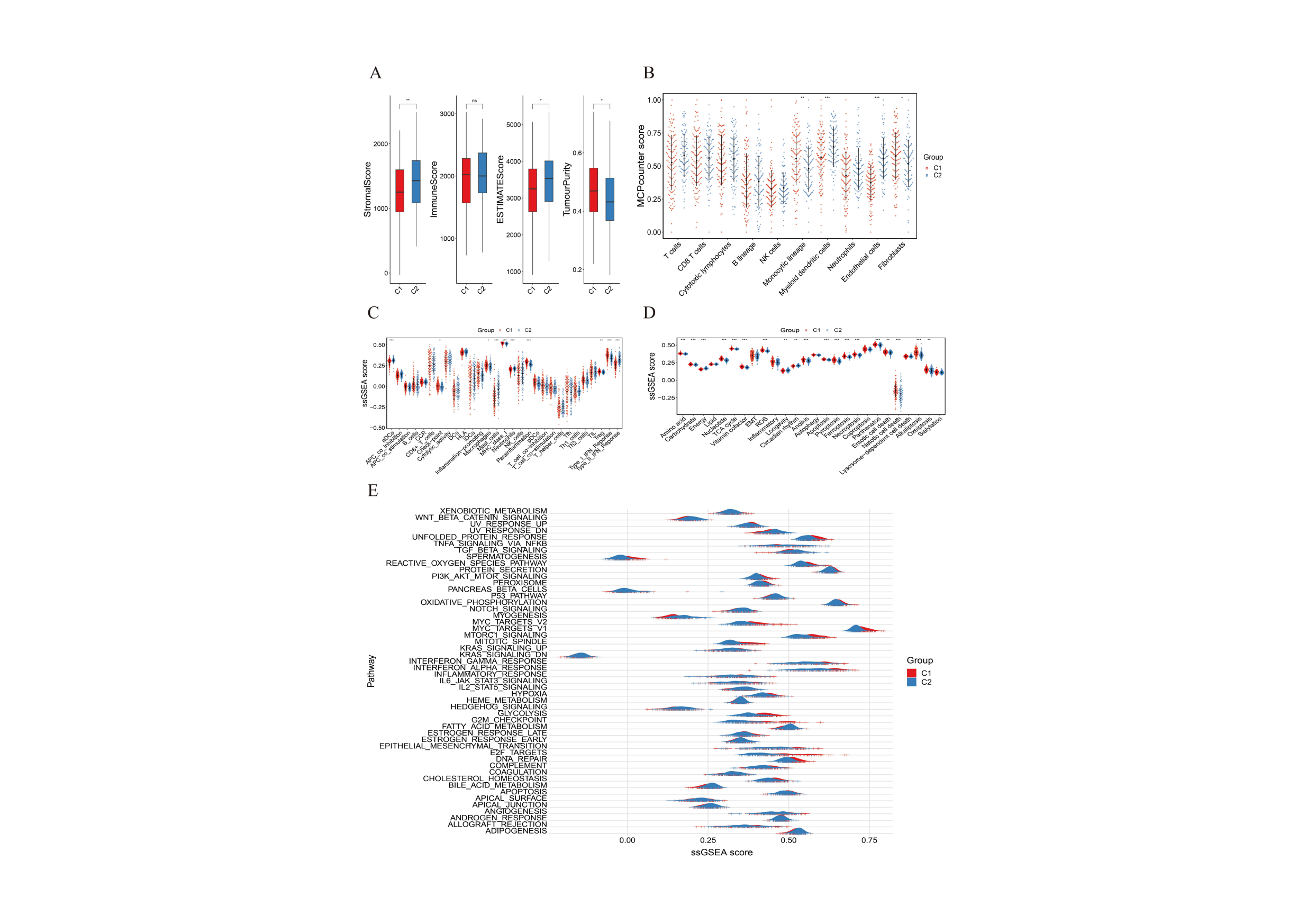


**Figure S2.** Differences of immunoinfiltration levels and pathways activities in GSE31210. **(A)** Box plots of TumourPurity score, StromalScore, ImmuneScore, and ESIMATEScore in C1 and C2. **(B)** MCPcounter scores and **(C)** ssGSEA scores were calculated to characterize the infiltrating levels of immune cells and related processes. ssGSEA scores of **(D)** metabolism-related pathways and **(E)** classical hallmark pathways of cancers in two subgroups. * *P* < 0.05, ** *P* < 0.01, *** *P* < 0.001.
