# Supplementary material for: The prognostic value of sialylation-related long non-coding RNAs in lung adenocarcinoma
Source: Sci Rep. 2024 Apr 17;14:8879. doi: 10.1038/s41598-024-59130-3 (PMC11024174; doi:10.1038/s41598-024-59130-3)
Supplement: Supplementary file 2 — Supplementary Information 2. [file 41598_2024_59130_MOESM2_ESM.pdf]

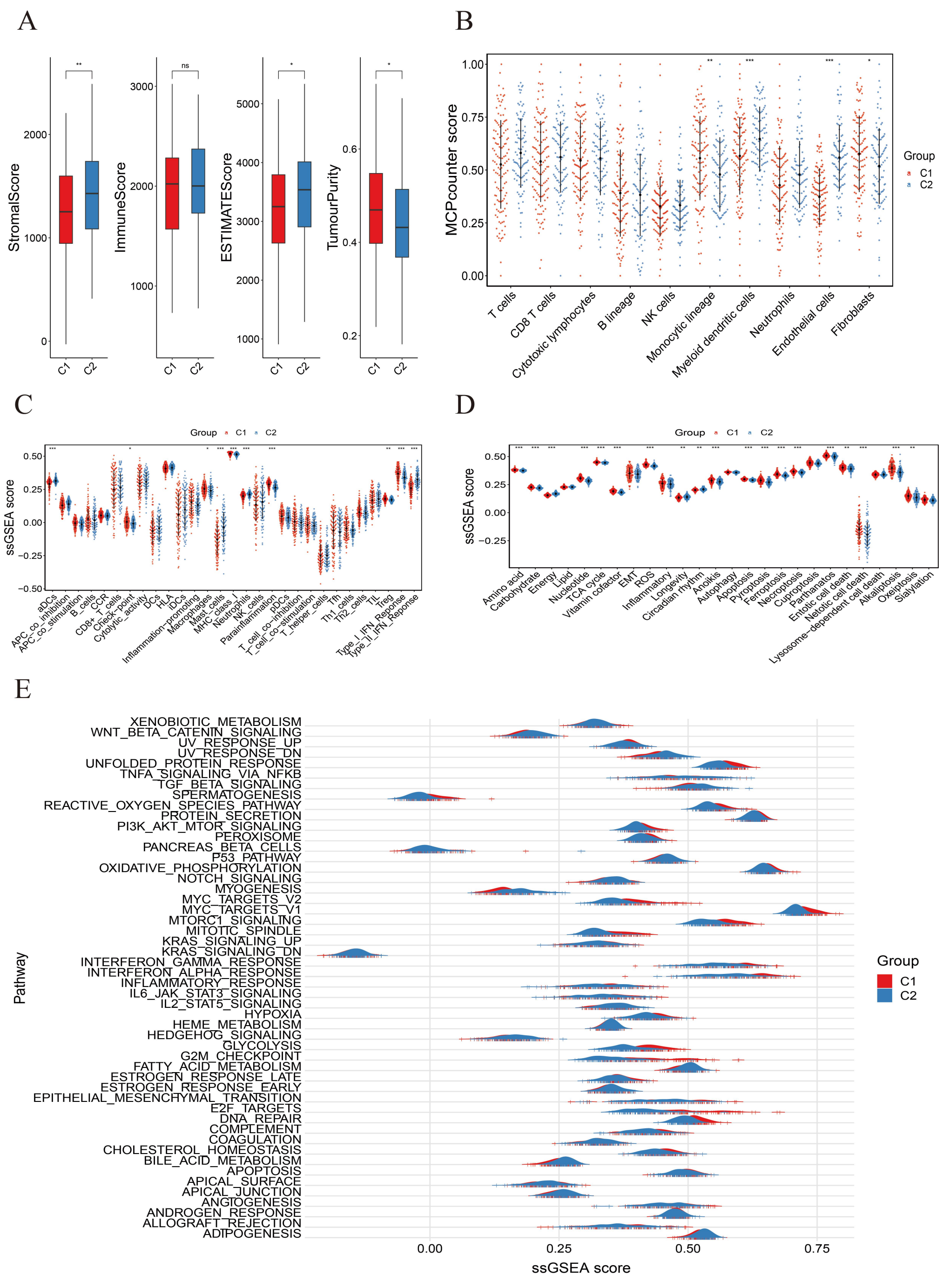

A
StromalScore
ImmuneScore
ESTIMATEScore
TumourPurity
C1
C2
B
MCPcounter score
Group
C1
C2
T cells
CD8 T cells
Cytotoxic lymphocytes
B lineage
NK cells
Monocytic lineage
Myeloid dendritic cells
Neutrophils
Endothelial cells
Fibroblasts
C
ssGSEA score
D
ssGSEA score
E
Pathway
ssGSEA score
XENOBIOTIC_METABOLISM
WNT_BETA_CATENIN_SIGNALING
UV_RESPONSE_UP
UV_RESPONSE_DN
UNFOLDED_PROTEIN_RESPONSE
TNFA_SIGNALING_VIA_NFKB
TGF_BETA_SIGNALING
SPERMATOGENESIS
REACTIVE_OXYGEN_SPECIES_PATHWAY
PROTEIN_SECRETION
PI3K_AKT_MTOR_SIGNALING
PEROXISOME
PANCREAS_BETA_CELLS
P53_PATHWAY
OXIDATIVE_PHOSPHORYLATION
NOTCH_SIGNALING
MYOGENESIS
MYC_TARGETS_V2
MYC_TARGETS_V1
MTORC1_SIGNALING
MITOTIC_SPINDLE
KRAS_SIGNALING_UP
KRAS_SIGNALING_DN
INTERFERON_GAMMA_RESPONSE
INTERFERON_ALPHA_RESPONSE
INFLAMMATORY_RESPONSE
IL6_JAK_STAT3_SIGNALING
IL2_STAT5_SIGNALING
HYPOXIA
HEME_METABOLISM
HEDGEHOG_SIGNALING
GLYCOLYSIS
G2M_CHECKPOINT
FATTY_ACID_METABOLISM
ESTROGEN_RESPONSE_LATE
ESTROGEN_RESPONSE_EARLY
EPITHELIAL_MESENCHYMAL_TRANSITION
E2F_TARGETS
DNA_REPAIR
COMPLEMENT
COAGULATION
CHOLESTEROL_HOMEOSTASIS
BILE_ACID_METABOLISM
APOPTOSIS
APICAL_SURFACE
APICAL_JUNCTION
ANGIOGENESIS
ANDROGEN_RESPONSE
ALLOGRAFT_REJECTION
ADIPOGENESIS
Group
C1
C2
